# Channel-Free Micro-Well–Template-Assisted Magnetic Particle Trapping for Efficient Single-Particle Isolation

**DOI:** 10.3390/mi16121397

**Published:** 2025-12-11

**Authors:** Jin-Yeong Park, Kyeong-Taek Nam, Young-Ho Nam, Yong-Kweon Kim, Seung-Ki Lee, Jae-Hyoung Park

**Affiliations:** 1 Department of Foundry Engineering, Dankook University, Yongin 16890, Republic of Korea; qkrwlsdud0921@nate.com; 2 Department of Electrical and Computer Engineering, Seoul National University, 1 Gwanak-ro, Gwanak-gu, Seoul 08826, Republic of Korea; kainosing11@snu.ac.kr (K.-T.N.); yongkkim@snu.ac.kr (Y.-K.K.); 3 Department of Electronics and Electrical Engineering, Dankook University, Yongin 16890, Republic of Korea; nyh7241@gmail.com; 4 Department of Semiconductor Convergence Engineering, Dankook University, Yongin 16890, Republic of Korea

**Keywords:** magnetic particle, particle trapping, dual surface

## Abstract

This study presents a channel-free, micro-well–template-assisted magnetic particle trapping method for efficient single-particle isolation without the need for microfluidic channels. Dual-surface silicon micro-well arrays were fabricated using photolithography, PE-CVD, and DRIE processes, featuring hydrophilic well interiors and hydrophobic outer surfaces to enhance trapping performance. The proposed method combines magnet-assisted sedimentation with rotational sweeping of a glass slide placed above the micro-well array, enabling rapid and uniform particle confinement within a 250 × 250 well array. Experimental results showed that the trapping efficiency increased with the well width and depth, achieving over 93.8% within three trapping cycles for optimized structures. High single-particle occupancy was obtained for wells of comparable size to the particle diameter, while deeper wells enabled stable trapping with minimal loss. The entire trapping process was completed within five minutes per cycle, demonstrating a rapid, simple, and scalable approach applicable to digital immunoassay systems for ultrasensitive biomolecule detection.

## 1. Introduction

The study of individually isolating and assembling colloidal microparticles on solid substrates has attracted considerable attention due to its wide range of applications in biosensing, digital immunoassays, and nanofabrication [1,2,3,4,5]. Recently, with the growing research interest in digital immunoassay techniques, this field has regained significant attention. Digital immunoassays typically employ microparticles to capture biological targets, followed by isolating and arranging these particles as individual entities. Subsequently, they are conjugated with specific detection reagents to amplify the signal, thereby enabling the detection of molecular-level signals. Such digital immunoassays have emerged as a powerful technique capable of detecting ultralow concentrations of biomolecules, allowing single-molecule quantification at the femtomolar or even lower levels [6,7,8,9]. These ultrasensitive analytical methods are essential for the early diagnosis of diseases such as cancer, neurodegenerative disorders, and infectious diseases, where conventional assays often fail to provide sufficient sensitivity.

Despite recent advances, achieving reliable single-particle isolation remains a significant challenge. Conventional approaches such as random deposition, droplet encapsulation, and micro-well filling each have inherent limitations. The method of randomly dispersing particles by dispensing and drying a droplet on a substrate offers the advantages of simplicity and ease of implementation compared with other techniques. However, as particle concentration increases, it becomes difficult to control aggregation and monolayer formation, often resulting in particle clustering or multilayer stacking. These issues hinder its applicability to highly sensitive digital immunoassay platforms [10,11]. This challenge primarily stems from the fact that, in conventional droplet or well-based deposition methods, evaporation-driven flows and interfacial tension gradients induce nonuniform particle transport along the air-liquid interface. These interfacial instabilities promote capillary-driven aggregation and multilayer particle stacking, rather than uniform spreading. As a result, achieving a true monolayer distribution becomes increasingly difficult, particularly at high bead concentrations where interparticle interactions are more pronounced [12,13,14,15]. In addition to these deposition-based limitations, existing magnetic and microfluidic trapping platforms for digital immunoassays typically combine enclosed microchannels, pressure or vacuum driven flow, and oil sealing with magnetic bead manipulation to achieve high bead loading and readout efficiency [16,17,18]. As a consequence, bead trapping behavior is largely governed by global flow fields and magnet configurations, and the specific influence of micro-well geometry or iterative seeding protocols on single-particle occupancy has not been systematically isolated or quantified [19]. Droplet-based methods, in which individual microparticles are encapsulated within droplets surrounded by oil, allow effective isolation of single particles. Nevertheless, the encapsulation efficiency is inherently limited by Poisson statistics, and additional flow control modules are required to improve yield [20,21,22]. In the micro-well approach, an aqueous solution containing microparticles is introduced onto a substrate, followed by the injection of oil or air to displace the solution and capture single particles within each micro-well [6,7,23,24,25,26]. Although this micro-well–based method can achieve high-density trapping, it relies on complex fluidic systems and precise flow control, thereby limiting its scalability and practical use in clinical applications.

In this study, a channel-free, micro-well–template-assisted single-particle trapping method employing magnetic particles is proposed to overcome the aforementioned challenges. Magnetic particles offer particularly attractive features for digital immunoassays, as they can be functionalized to selectively bind target biomolecules and can be rapidly manipulated using external magnetic fields [27]. Within this framework, we integrate three synergistic design strategies to address the aforementioned limitations: magnet-assisted sedimentation, a dual-surface micro-well architecture, and controlled seeding cycles. First, the external magnetic field rapidly pulls the magnetic particles toward the substrate prior to significant droplet evaporation, thereby suppressing evaporation-driven interfacial flows and edge-directed accumulation that typically lead to aggregation [28,29,30]. Second, the engineered combination of hydrophilic well interiors and hydrophobic surrounding surfaces promotes preferential particle localization into the wells during the drying and rotational sweeping steps, effectively converting interfacial forces into a selective confinement mechanism [14,15,31]. Third, by precisely tuning the geometric ratios of well width and depth to particle diameter together with the number of seeding cycles, we establish a trapping regime in which single-particle occupancy is mechanically favored over multi-particle cluster formation [32,33,34,35]. Through these combined mechanisms, the proposed platform effectively suppresses uncontrolled aggregation while enabling uniform, size-selective single-particle isolation that is fully compatible with digital immunoassay workflows. These characteristics enable the rapid and efficient separation of biomarkers even in complex biological fluids. Moreover, magnetic beads are already widely employed in commercial immunoassay kits and nucleic acid purification platforms, further highlighting their practical relevance. However, efficient trapping and alignment of magnetic particles within micro-wells without the aid of fluidic channels have not been sufficiently investigated. In the proposed approach, an array of 250 × 250 micro-wells is fabricated on a substrate within a compact 1.25 mm × 1.25 mm area to achieve high density isolation and ordered arrangement of particles. A small volume (2 µL) of particle suspension is dispensed onto the template, followed by magnetic-assisted sedimentation, removal of the supernatant, drying, and rotational sweeping steps. The entire process is completed within five minutes without the need for syringe pumps or microfluidic channels. Micro-wells of various dimensions were also fabricated to evaluate trapping performance across different particle sizes.

## 2. Materials and Methods

### 2.1. Fabrication

**Figure 1:**
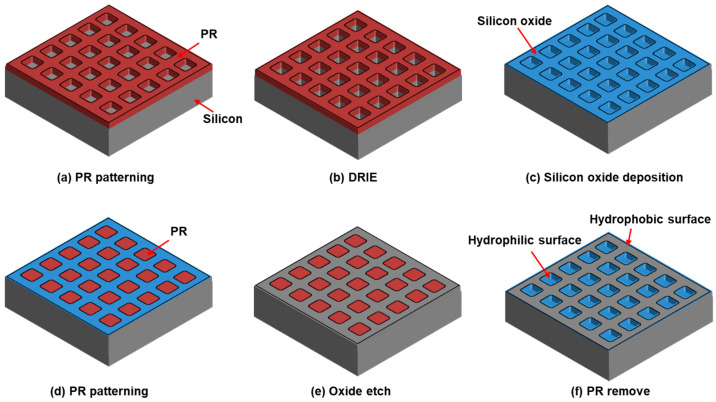
Fabrication process for the dual-surface silicon micro-well array structures.

Figure 1 presents a schematic illustration of the fabrication process for the silicon micro-well structures. As a standard cleaning step, a silicon wafer was immersed in an SPM solution (H_2_SO_4_:H_2_O_2_ = 4:1) for 20 min, followed by a 10 min quick dump rinse (QDR) and spin drying. After dehydration, hexamethyldisilazane (HMDS) was applied as an adhesion promoter, and a positive photoresist (SS03A9, Dongwoo Fine-Chem, Pyeongtaek, Republic of Korea) was spin-coated at 4000 rpm for 35 s, yielding a thickness of approximately 0.8 μm. The wafer was then soft-baked at 88 °C for 60 s, and an array of 250 × 250 square wells with designed lateral dimensions of 1.5 μm, 2.0 μm, and 2.5 μm was patterned using a maskless lithography system (DL-1000 HP, NanoSystem Solutions, Okinawa, Japan) (Figure 1a). The exposed silicon was vertically etched using a deep reactive ion etching (DRIE) process (SLR-770-10R-B, Plasma Therm, Petersburg, FL, USA) with the photoresist serving as an etch mask (Figure 1b). The etch depth was controlled through the DRIE process cycles to define the well structures. The residual photoresist was removed by two consecutive SPM cleaning steps. Subsequently, a 100 nm-thick SiO_2_ layer was deposited by plasma-enhanced chemical vapor deposition (PECVD, PlasmaPro 100, Oxford Instruments, Abingdon, UK) (Figure 1c). To form dual surface regions, a negative photoresist (DNRL-300) was spin-coated to a thickness of approximately 2 μm and soft-baked at 90 °C for 60 s, followed by patterning such that the resist remained only inside the etched wells (Figure 1d). Reactive ion etching (RIE, NeoS-MAXIS 200L, Gigalane, Hwaseong, Republic of Korea) was then used to selectively remove the oxide layer from the outer regions (Figure 1e), and the remaining resist was completely stripped via an additional SPM cleaning step (Figure 1f). Finally, the silicon wafer was diced into 1 cm × 1 cm chips using a dicing saw (DAD3221, DISCO Corporation, Tokyo, Japan). The resulting structure exhibited hydrophilic silicon oxide inside the micro-wells and hydrophobic silicon on the outer surfaces. This dual-surface configuration was designed to enhance trapping efficiency during the magnetic particle capture process within the micro-wells.

### 2.2. Magnetic Particle Trapping Method

**Figure 2:**
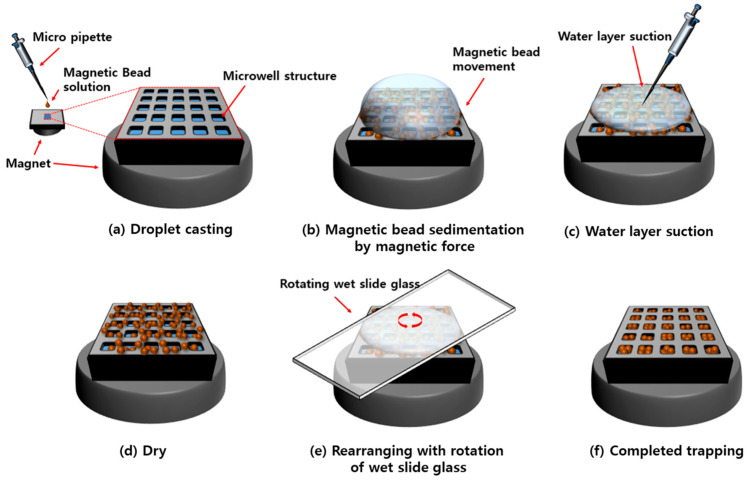
Schematic illustration of the channel-free magnetic particle trapping process using a dual-surface micro-well array template.

Figure 2 illustrates the channel-free magnetic particle trapping method using a micro-well array structure. Two types of magnetic particles were employed in the experiments: 1.00 μm (CM-10-10, Spherotech, Lake Forest, IL, USA) and 1.88 μm (CM-10-15, Spherotech, Lake Forest, IL, USA) in diameter. The 1.00 μm magnetic particles were diluted in an ethanol mixture (ethanol/deionized water = 8:2) to a concentration of 2.75 × 10^9^ particles/mL, while the 1.88 μm magnetic particles were diluted in an ethanol mixture (ethanol/deionized water = 5:5) to a concentration of 5.0 × 10^9^ particles/mL.

A cylindrical neodymium magnet with a diameter of 7 mm and a height of 2 mm was positioned beneath the diced 1 cm × 1 cm micro-well chip. A 2 μL droplet of the diluted magnetic particle suspension was dispensed onto the micro-well array using a micropipette (Figure 2a). Under the influence of the magnetic field, the magnetic beads sedimented downward and accumulated at the bottom, leaving only the ethanol mixture in the upper layer (Figure 2b). Magnetic-assisted sedimentation minimizes the coffee-ring effect [1] that typically occurs during the drying of ethanol mixtures and promotes uniform distribution of magnetic particles across the template surface. Subsequently, the supernatant was carefully removed with a micropipette to reduce the drying time (Figure 2c). The template was then air-dried at room temperature for 2 min to ensure complete evaporation of the remaining liquid (Figure 2d). The dried template, along with the magnet, was fixed at the center of a rotary mixing device (ThermoMixer C, Eppendorf, Hamburg, Germany). A 4 cm × 2.5 cm slide glass was wetted with deionized water and gently placed on top of the template. The mixer was rotated at 800 rpm for 60 s. During rotation, the slide glass rotated more slowly than the mixer platform, with its rotational speed measured at 34 ± 2 rpm (Figure 2e). As the slide glass rotated, the concentrated magnetic particles were guided into individual micro-wells, while untrapped particles were displaced outward from the template surface. After the rotation step, the slide glass was removed, and the sample was dried for 1 min. This sequence constituted one trapping cycle, and the process was repeated to achieve complete filling of the micro-wells with magnetic particles (Figure 2f).

During droplet deposition, spatially non-uniform evaporation generates interfacial flows that drive particles toward the droplet edge or induce local aggregation within the thinning liquid film [12,13,31]. To suppress such lateral transport, an external magnetic field is applied immediately after dispensing, rapidly drawing the magnetic particles vertically toward the substrate and thereby minimizing evaporation induced drift. Once the particles are uniformly sedimented, a rotational sweeping step is used to redistribute them laterally across the surface. During this process, the wettability contrast hydrophilic micro-well interiors surrounded by hydrophobic regions creates energetically favorable confined wetting domains within the wells, while unstable particles located outside the wells are effectively removed by shear during sweeping [31,32,33,34]. The coupled actions of magnetic settling, sweeping-assisted lateral sorting, and wettability-guided confinement collectively establish the physical mechanism that enables stable, size selective particle trapping in the micro-well array. The magnetic particle trapping process using the template was a rapid operation, completed within 5 min per trapping cycle, and the capture yield was further improved through multiple repetitions of the trapping procedure. All experiments were conducted at room temperature, and the particle-trapping results within individual micro-wells were observed using an optical microscope (BX53, Olympus, Tokyo, Japan) and a field-emission scanning electron microscope (FE-SEM, S-4700, Hitachi, Tokyo, Japan). For quantitative evaluation, the trapping efficiency was determined by averaging the measurements from five non-overlapping regions of interest, each comprising 50 × 50 wells (2500 wells), sampled from the center, top, bottom, left, and right areas of the 250 × 250 micro-well array. Single-particle occupancy was evaluated under the same measurement conditions used for trapping efficiency.

## 3. Results and Discussion

### 3.1. Fabrication Results

**Figure 3:**
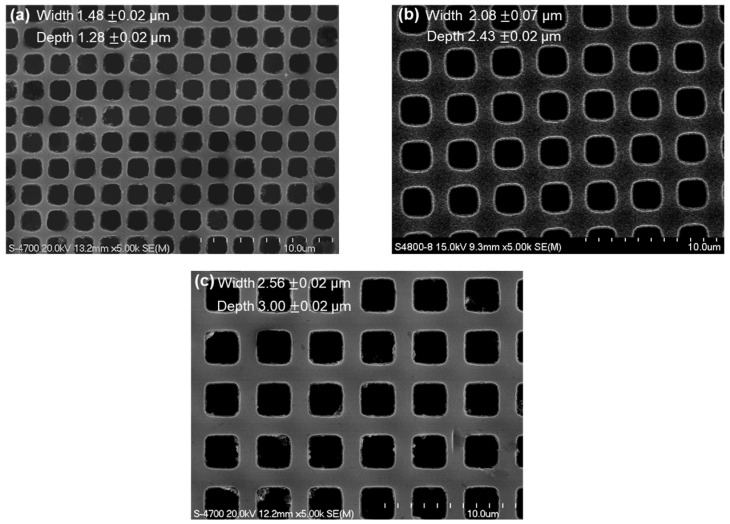
FE-SEM images of the fabricated silicon micro-well array templates with different lateral dimensions and depths. (**a**) Micro-wells with a diameter of 1.48 ± 0.02 μm and a depth of 1.28 ± 0.02 μm. (**b**) Micro-wells with a diameter of 2.08 ± 0.07 μm and a depth of 2.43 ± 0.02 μm. (**c**) Micro-wells with a diameter of 2.56 ± 0.02 μm and a depth of 3.00 ± 0.02 μm.

Figure 3 shows FE-SEM images of the fabricated micro-well array template structures. The measured lateral dimensions of the silicon wells were 1.48 ± 0.02 μm (Figure 3a), 2.08 ± 0.07 μm (Figure 3b), and 2.56 ± 0.02 μm (Figure 3c), corresponding to depths of 1.28 ± 0.02 μm, 2.43 ± 0.02 μm, and 3.00 ± 0.02 μm, respectively. These results confirm the reproducible fabrication of precisely defined micro-well arrays suitable for efficient particle trapping.

### 3.2. Magnetic Particle Trapping Results

**Figure 4:**
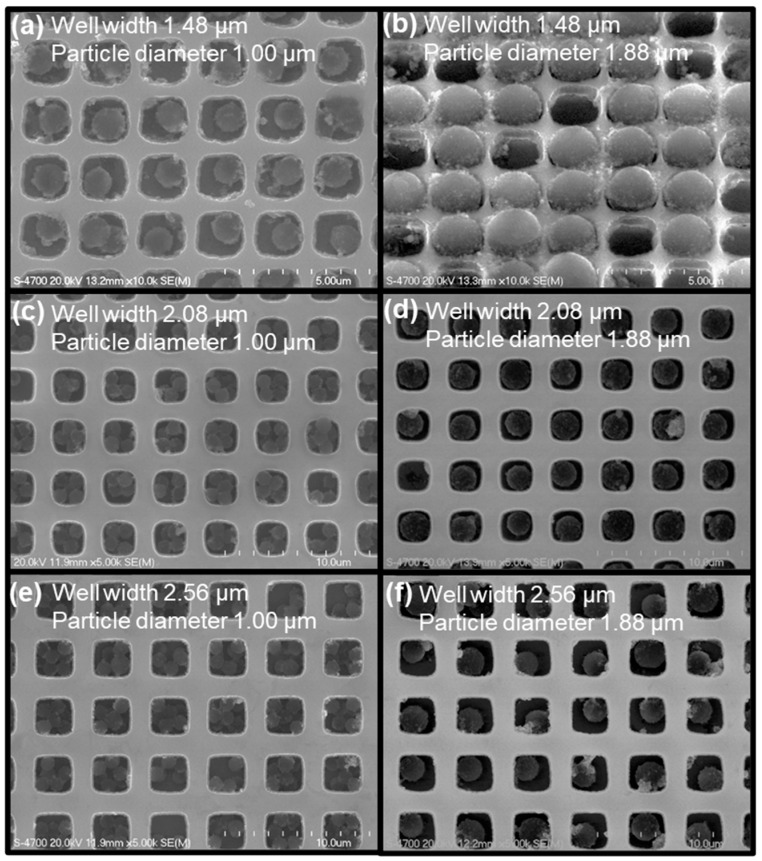
FE-SEM images showing the trapping behavior of magnetic particles within the fabricated silicon micro-well arrays under different well dimensions and particle sizes: (**a**) 1.00 μm magnetic particles trapped in 1.48 μm-wide wells, (**b**) 1.88 μm magnetic particles trapped in 1.48 μm wells, (**c**) 1.00 μm magnetic particles trapped in 2.08 μm wells, (**d**) 1.88 μm magnetic particles trapped in 2.08 μm wells, (**e**) 1.00 μm magnetic particles trapped in 2.56 μm wells, (**f**) 1.88 μm magnetic particles trapped in 2.56 μm wells.

The trapping performance of the fabricated micro-well arrays was evaluated using 1.00 μm and 1.88 μm magnetic particles, as shown in the FE-SEM images in Figure 4. Figure 4a shows 1.00 μm magnetic particles trapped in micro-wells with a width of approximately 1.48 μm, where one to three particles were captured within individual micro-wells. Figure 4b presents 1.88 μm magnetic particles trapped in 1.48 μm wells, with each well typically containing a single particle positioned on top of the structure due to its larger size relative to the well width. Figure 4c shows 1.00 μm magnetic particles trapped in 2.08 μm wells, where most wells contained clusters of four or more particles. In contrast, Figure 4d illustrates 1.88 μm magnetic particles captured in 2.08 μm wells, demonstrating that the comparable dimensions of the wells and particles allowed them to fit snugly within the cavities. Figure 4e displays 1.00 μm magnetic particles trapped in 2.56 μm wells, where no single-particle trapping was observed and most wells contained clusters of multiple particles. Finally, Figure 4f shows 1.88 μm magnetic particles trapped in 2.56 μm wells, achieving over 99% single-particle occupancy, with only rare cases of two overlapping particles observed within the same well.

**Figure 5:**
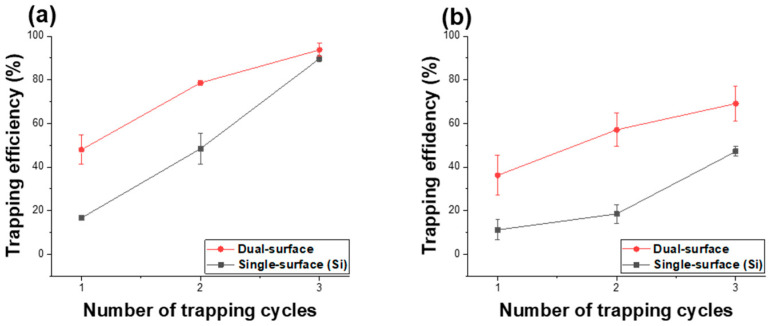
Trapping efficiency of magnetic particles versus number of trapping cycles for dual-surface and single-surface (Si) micro-well templates. (**a**) Trapping efficiency of 1.00 µm magnetic particles in micro-wells with a width of 1.48 µm, (**b**) Trapping efficiency of 1.88 µm magnetic particles in the same micro-well templates.

Figure 5 presents the trapping efficiency results obtained from the proposed channel-free magnetic particle capture experiment using a micro-well array template with a well width of 1.48 μm. Two types of templates were used for comparison: a dual-surface structure in which only the inner regions of the micro-wells were patterned with silicon oxide, and a single-surface structure composed entirely of silicon, including the well interiors. The trapping efficiency was evaluated over one to three trapping cycles for both structures. The trap rate was defined as the ratio of wells containing particles to the total number of wells. The number of micro-wells and the trapped particle counts were determined using image analysis with the ImageJ software (version 1.54i) . Figure 5a shows the trap rate for 1.00 μm magnetic particles. For the dual-surface structure, the trapping rates after one, two, and three cycles were measured as 48 ± 6.7%, 78.7 ± 1.2%, and 93.8 ± 3.0%, respectively, indicating a gradual increase in trapping efficiency with the number of trapping cycles. Although a similar trend was observed for the single-surface silicon micro-well array, the overall trapping efficiency was lower compared to the dual-surface template. This demonstrates that forming a hydrophilic surface only inside the micro-wells effectively confines the particles during the trapping process, thereby improving trapping efficiency. Among the particles trapped in the 1.48 μm wells, 90 ± 7.2% were captured as single particles, while double and triple particle trapping occurred at rates of 8.8 ± 5.6% and 2.2 ± 1.5%, respectively. Figure 5b shows the trapping efficiency results for 1.88 μm magnetic particles captured in micro-wells with a width of 1.48 μm for both the dual-surface and single-surface templates. Since the particle diameter was larger than the well width, single-particle trapping was dominant regardless of the number of trapping cycles. In the dual-surface structure, the trapping efficiency increased from 36.2 ± 9.1% after one cycle to 69.1 ± 8.0% after three cycles, whereas the single-surface silicon wells exhibited lower trapping efficiencies of 11.2 ± 4.6% and 47.1 ± 2.2% after one and three cycles, respectively. In this case, the particle size being larger than the well diameter resulted in a lower overall trapping efficiency compared with that observed for smaller particles.

**Figure 6:**
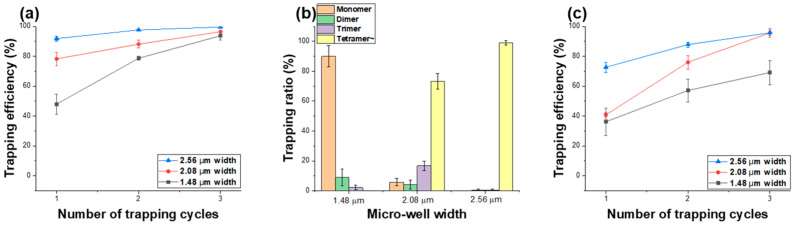
Trapping performance of magnetic particles as a function of micro-well size using dual-surface templates. (**a**) Trapping efficiency of 1.00 µm magnetic particles for micro-well widths of 1.48 µm, 2.08 µm, and 2.56 µm after one to three trapping cycles, (**b**) Distribution of trapping ratios showing the proportion of wells containing one (monomer), two (dimer), three (trimer), or four (tetramer) 1.00 µm particles, depending on the micro-well width, (**c**) Trapping efficiency of 1.88 µm magnetic particles for the same three micro-well sizes.

Using the channel-free magnetic particle trapping method, the trapping efficiency and the number of particles captured per individual micro-well were evaluated for dual-surface micro-well structures with widths of 1.48 ± 0.02 μm, 2.08 ± 0.07 μm, and 2.56 ± 0.02 μm, as shown in Figure 6. Figure 6a presents the trapping efficiency of 1 μm magnetic particles for the three different template sizes. When a single trapping cycle was performed, the trapping efficiency increased by increasing well size, indicating that larger micro-wells enhance particle capture. As the number of trapping cycles increased to three, the trapping efficiency reached saturation, showing over 93% for all well sizes regardless of template dimensions. Figure 6b shows the comparison of trapping ratios for different numbers of 1 μm particles captured per individual micro-well, depending on the micro-well size. For the 1.48 μm micro-well structure, the ratios of monomer, dimer, and trimer trapping were measured as 90.00 ± 7.2%, 8.88 ± 5.60%, and 2.22 ± 1.57%, respectively. Because the particle size was comparable to the well width, most trapping occurred as single particles, while the few dimers and trimers observed were stacked vertically within the wells. In contrast, as the micro-well size increased to 2.08 μm, the fraction of wells containing multiple particles increased significantly, with tetramer trapping reaching 73.3 ± 5.13%. For the larger 2.56 μm micro-well template, no single-particle trapping was observed, and the distribution was measured as 0.5 ± 0.67% for dimers, 0.5 ± 0.67% for trimers, and 99 ± 1.34% for tetramers, indicating that nearly all wells contained clusters of four or more particles. Figure 6c compares the trapping efficiencies of 1.88 μm magnetic particles captured using the same three micro-well templates. In this case as well, the trapping efficiency increased with increasing well size. However, for the 1.48 μm template, since the particle diameter (1.88 μm) was larger than the well width, the particles were trapped while resting across the top of the wells rather than being fully confined inside. Consequently, even after three trapping cycles, the overall trapping efficiency remained below 90%, lower than that of the larger templates. For the 1.48 μm and 2.08 μm micro-well structures, 1.88 μm magnetic particles were captured exclusively as single particles (100% single-particle trapping), while in the 2.56 μm wells, a single-particle trapping ratio of approximately 99% was observed, with only a few cases showing slight particle aggregation. Spatial uniformity was assessed by comparing trapping efficiencies across five positions of the array (center, top, bottom, left, and right). The measured values showed no meaningful positional variation (1.00-µm beads: 93.8 ± 3.0% center vs. 92.3–94.1% edges; 1.88-µm beads: 92.3 ± 4.2% center vs. 91.0–93.3% edges), confirming that the trapping performance is uniform across the entire array.

**Figure 7:**
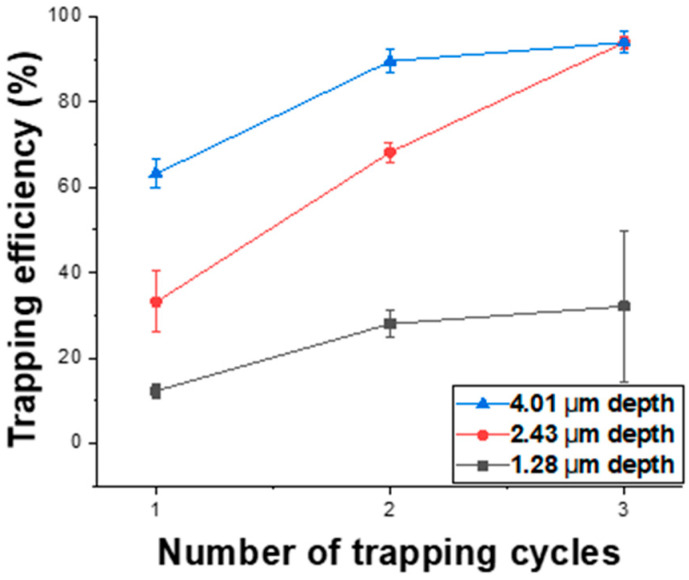
Trapping efficiency of 1.88 µm magnetic particles as a function of micro-well depth and the number of trapping cycles.

**Figure 8:**
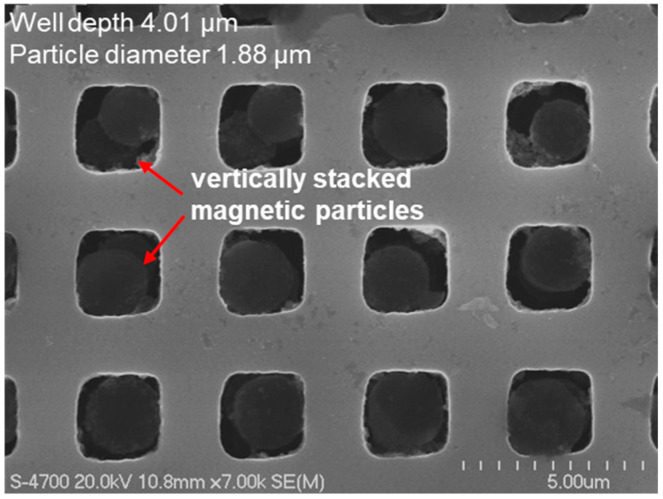
FE-SEM image showing magnetic particles trapped in deep micro-wells (depth: 4.01 ± 0.02 μm).

Figure 7 shows the trapping efficiency of magnetic particles as a function of micro-well depth. Templates consisting of micro-wells with an approximate width of 2 μm and varying depths of 1.28 ± 0.02 μm, 2.43 ± 0.02 μm, and 4.01 ± 0.02 μm were fabricated to measure and compare the trapping efficiencies of 1.88 μm magnetic particles under identical experimental conditions. The results indicate that deeper micro-wells exhibit higher trapping efficiencies even at the same number of trapping cycles. As the number of trapping cycles increased, the trapping efficiency also improved, reaching 94.0 ± 1.45% and 94.0 ± 2.60% after three trapping cycles for the 2.43 μm- and 4.01 μm-deep micro-well templates, respectively. This demonstrates that micro-wells with widths and depths larger than the particle size achieve very high trapping efficiencies when three or more trapping cycles are performed. In contrast, for the shallower micro-wells with a depth of 1.28 μm—smaller than the particle diameter—the trapping efficiency increased slightly with additional trapping cycles but remained relatively low at 32.14 ± 17.6% after three cycles, with a larger variation observed across repeated experiments. This reduced efficiency is likely due to the insufficient well depth, which increases the probability of particles being displaced from the wells during the rotational motion of the slide glass. For the deeper micro-wells (4.01 μm), whose depth was considerably greater than the particle size, high trapping efficiencies were achieved even with only one or two trapping cycles. However, as shown in the SEM images in Figure 8, some wells contained two vertically stacked magnetic particles, in addition to single-particle trapping. After three trapping cycles, the proportion of wells containing two stacked particles was measured to be approximately 75.00% of the total trapped wells.

The observed dependence of trapping behavior on well width (W), well depth (D), and particle size (P) can be interpreted through the combined effects of geometric confinement and interfacial forces [2,3,33,34]. When the well width is comparable to the particle diameter (W ≈ P), lateral confinement restricts side by side aggregation, resulting in predominantly single-particle occupancy. As W increases relative to P, the available cross-sectional area becomes sufficient to host multiple particles, thereby increasing the likelihood of multi-particle cluster formation; this trend is reflected in the high fraction of tetramer trapping observed for 1.00 µm beads in 2.56 µm wide wells. Similarly, when the well depth is smaller than the particle diameter (D < P), vertical confinement is insufficient, and particles are more susceptible to displacement by shear and torque generated during the rotational sweeping step, leading to reduced trapping efficiency and greater experimental variability. In contrast, for wells with depths greater than the particle diameter (D > P), enhanced vertical confinement enables stable particle retention across repeated trapping cycles, although excessively deep wells may promote vertical stacking. These findings demonstrate that the proposed channel-free trapping scheme can be systematically optimized by tuning the geometric ratios of well width and depth to particle diameter (W/P and D/P) to achieve a balance between high trapping efficiency and high single-particle occupancy, representing a mechanistic improvement over previously reported micro-well–based loading approaches.

Collectively, these results demonstrate that optimal single-particle trapping is achieved within a well-defined geometric window rather than by merely satisfying the condition D/P ≥ 1. Stable vertical confinement without undesired particle stacking was obtained when the well depth to particle diameter ratio (D/P) was maintained between approximately 1.0 and 1.3. Similarly, high single-particle occupancy was consistently achieved when the well width-to-particle diameter ratio (W/P) was kept within the range of ~1.3–1.5, which effectively suppressed lateral clustering. This optimized geometric regime aligns with our experimental observations for 1.88-µm beads in wells with W = 2.56 µm and D = 2.4–3.0 µm, which exhibited the most stable and size-selective single-particle trapping performance.

In digital immunoassays, immunochemical reactions are generally performed in the bulk solution prior to micro-well loading to avoid mass transport constraints and to minimize surface-induced nonspecific adsorption [8,36,37,38]. Following this established workflow, magnetic particles can be functionalized, incubated with target analytes, and subsequently labeled with optical, enzymatic, or plasmonic reporters before introduction into the micro-well array. Once loaded, magnet assisted sedimentation combined with geometric confinement enables stable single particle isolation, allowing each micro-well to serve as an independent reaction or detection compartment. Enzyme based assays can be carried out by adding fluorogenic substrates after trapping, whereas SERS-based detection planned for future studies can be implemented using particles prelabeled with SERS- active nanotags. Collectively, these considerations demonstrate that the proposed trapping platform is fully compatible with practical digital immunoassay workflows. In addition, nonspecific bead adhesion on the substrate is inherently minimized by the wettability contrast of the dual-surface design [33,34]. Beads that are not securely confined within the hydrophilic micro-wells experience unfavorable interfacial interactions on the surrounding hydrophobic surfaces and are subsequently removed during the rotational sweeping step [31,33]. This process yields a naturally low-background trapping environment that is well suited for bead-based digital assay applications. For the application of such particle-trapping micro-well array templates to digital immunoassay technologies, it is essential to achieve high single-particle trapping efficiency within individual micro-wells. In the trapping method proposed in this study, a high single-particle trapping yield can be achieved simply by optimizing the width and depth of the micro-wells. The proposed channel-free micro-well trapping approach, which integrates a dual-surface micro-well structure with magnet-assisted sedimentation and rotational sweeping of the slide glass above the wells, achieved a high trapping efficiency exceeding 90% across a 250 × 250 micro-well arrays within three trapping cycles.

## 4. Conclusions

In this study, we introduced a dual-surface micro-well structure and a channel-free magnetic particle trapping method. Micro-well structures with widths of 1.48 ± 0.02 μm, 2.08 ± 0.07 μm, and 2.56 ± 0.02 μm were fabricated using photolithography, PE-CVD, and DRIE processes. The trapping performance was evaluated under various conditions using two types of magnetic particles with diameters of 1.00 μm and 1.88 μm. By employing a dual-surface micro-well design—where only the inner regions were fabricated as hydrophilic surfaces, the trapping efficiency was significantly enhanced. The proposed channel-free, magnet-assisted trapping method enabled fast and efficient single-particle capture without the need for complex microfluidic channels. As a result, a trapping efficiency exceeding 93.8% was achieved across a 250 × 250 micro-well arrays. Future work will focus on optimizing the rotational speed of the slide glass above the micro-wells to ensure stable trapping of biomolecule-conjugated particles and extending this technique for precise single-particle confinement. Ultimately, the developed method is expected to be applied to digital immunoassay platforms for ultrasensitive detection of biomolecules based on single-particle analysis.

## Data Availability

The original contributions presented in this study are included in the article. Further inquiries can be directed to the corresponding author.

## References

[1] Agarwal G., Servi A., Eid F., Livermore C. (2010). Selective self-assembly of polymer structures using templated assembly by selective removal. IEEE Trans. Nanotechnol..

[2] Malaquin L., Kraus T., Schmid H., Delamarche E., Wolf H. (2007). Controlled particle placement through convective and capillary assembly. Langmuir.

[3] Pioli R., Fernández-Rodríguez M.A., Grillo F., Álvarez L., Stocker R., Isa L., Secchi E. (2021). Sequential capillarity-assisted particle assembly in a microfluidic channel. Lab Chip.

[4] Winkleman A., Gates B.D., McCarty L.S., Whitesides G.M. (2005). Directed self-assembly of spherical particles on patterned electrodes by an applied electric field. Adv. Mater..

[5] Prevo B.G., Hwang D.K., Zacharia N.S., Prasad V., Velev O.D. (2007). Engineered deposition of coatings from nano- and micro-particles: A brief review of convective assembly at high volume fraction. Colloids Surf. A Physicochem. Eng. Asp..

[6] Rissin D.M., Kan C.W., Campbell T.G., Howes S.C., Fournier D.R., Song L., Piech T., Patel P.P., Chang L., Rivnak A.J. (2010). Single-molecule enzyme-linked immunosorbent assay detects serum proteins at subfemtomolar concentrations. Nat. Biotechnol..

[7] Kim S.H., Iwai S., Araki S., Sakakihara S., Iino R., Noji H. (2012). Large-scale femtoliter droplet array for digital counting of single biomolecules. Lab Chip.

[8] Zhang Y., Gu H., Xu H. (2024). Recent progress on digital immunoassay: How to achieve ultrasensitive, multiplex and clinical accessible detection?. Sens. Diagn..

[9] Su Y., Tian J., Zhou L. (2024). Review of single-molecule immunoassays: Non-chip and chip-based digital assays. Anal. Chim. Acta.

[10] Wu C., Garden P.M., Walt D.R. (2020). Ultrasensitive detection of attomolar protein concentrations by dropcast single molecule assays. J. Am. Chem. Soc..

[11] Wang W., Peng Y., Wu J., Zhang M., Li Q., Zhao Z., Liu M., Wang J., Cao G., Bai J. (2022). Ultrasensitive automatic detection of small molecules by membrane imaging of single molecule assays. ACS Appl. Mater. Interfaces.

[12] Hu H., Larson R.G. (2005). Analysis of the effects of Marangoni stress on the microflow in an evaporating sessile droplet. Langmuir.

[13] Yunker P.J., Still T., Lohr M.A., Yodh A.G. (2011). Suppression of the coffee-ring effect by shape-dependent capillary interactions. Nature.

[14] Grzelczak M., Vermant J., Furst E.M., Liz-Marzán L.M. (2010). Directed self-assembly of nanoparticles. ACS Nano.

[15] Xu G., Chen A., Feng F., Wu Y., Wang X. (2024). Multiscale Mass Transport Across Membranes: From Molecular Scale to Nanoscale to Micron Scale. ACS Nano.

[16] Sharma H., Yadav V., Burchett A., Shi T., Senapati S., Datta M., Chang H.-C. (2025). A Mem-dELISA platform for dual color and ultrasensitive digital detection of colocalized proteins on extracellular vesicles. Biosens. Bioelectron..

[17] Zandi Shafagh R., Decrop D., Ven K., Vanderbeke A., Hanusa R., Breukers J., Pardon G., Haraldsson T., Lammertyn J., van der Wijngaart W. (2019). Reaction injection molding of hydrophilic-in-hydrophobic femtolitre-well arrays. Microsyst. Nanoeng..

[18] Tripodi L., Ven K., Kil D., Rutten I., Puers R., Lammertyn J. (2018). Teflon-on-Glass Molding Enables High-Throughput Fabrication of Hydrophilic-in-Hydrophobic Microwells for Bead-Based Digital Bioassays. Materials.

[19] Curtin K., Fike B.J., Binkley B., Godary T., Li P. (2022). Recent Advances in Digital Biosensing Technology. Biosensors.

[20] Shim J., Ranasinghe R.T., Smith C.A., Ibrahim S.M., Hollfelder F., Huck W.T.S., Klenerman D., Abell C. (2013). Ultrarapid generation of femtoliter microfluidic droplets for single-molecule-counting immunoassays. ACS Nano.

[21] Cohen L., Cui N., Cai Y., Garden P.M., Li X., Weitz D.A., Walt D.R. (2020). Single-molecule protein detection with attomolar sensitivity using droplet digital enzyme-linked immunosorbent assay. ACS Nano.

[22] Yue X., Fang X., Sun T., Yi J., Kuang X., Guo Q., Wang Y., Gu H., Xu H. (2022). Breaking through the Poisson distribution: A compact high-efficiency droplet microfluidic system for single-bead encapsulation and digital immunoassay detection. Biosens. Bioelectron..

[23] Rissin D.M., Kan C.W., Campbell T.G., Howes S.C., Fournier D.R., Song L., Patel P.P., Chang L., Rivnak A.J., Patel P.P. (2011). Simultaneous detection of single molecules and singulated ensembles of molecules enables immunoassays with broad dynamic range. Anal. Chem..

[24] Witters D., Knez K., Ceyssens F., Puers R., Lammertyn J. (2013). Digital microfluidics-enabled single-molecule detection by printing and sealing single magnetic beads in femtoliter droplets. Lab Chip.

[25] Kan C.W., Rivnak A.J., Campbell T.G., Piech T., Rissin D.M., Mösl M., Peterca A., Niederberger H.-P., Minnehan K.A., Patel P.P. (2012). Isolation and detection of single molecules on paramagnetic beads using sequential fluid flows in microfabricated polymer array assemblies. Lab Chip.

[26] Kan C.W., Tobos C.I., Rissin D.M., Wiener A.D., Meyer R.E., Svancara D.M., Comperchio A., Warwick C., Millington R., Collier N. (2020). Digital enzyme-linked immunosorbent assays with sub-attomolar detection limits based on low numbers of capture beads combined with high efficiency bead analysis. Lab Chip.

[27] Yari P., Rezaei B., Dey C., Chugh V.K., Veerla N.V.R.K., Wang J.-P., Wu K. (2023). Magnetic particle spectroscopy for point-of-care: A review on recent advances. Sensors.

[28] Demirörs A.F., Johnson P.M., van Kats C.M., van Blaaderen A., Imhof A. (2010). Directed self-assembly of colloidal dumbbells with an electric field. Langmuir.

[29] Wang J., Wu X., Wang C., Shao N., Dong P., Xiao R., Wang S. (2015). Magnetically Assisted Surface-Enhanced Raman Spectroscopy for the Detection of Staphylococcus aureus Based on Aptamer Recognition. ACS Appl. Mater. Interfaces.

[30] Wang X., Feng X., Ma G., Yao L., Ge M. (2016). Amphiphilic Janus Particles Generated via a Combination of Diffusion-Induced Phase Separation and Magnetically Driven Dewetting and Their Synergistic Self-Assembly. Adv. Mater..

[31] Colosqui C.E., Morris J.F., Stone H.A. (2013). Hydrodynamically driven colloidal assembly in dip coating. Phys. Rev. Lett..

[32] Shillingford C., Lee D., Bertin V., Sauret A., Dressaire E. (2020). Capillary assembly of liquid particles. Small.

[33] Yu H.S.C., Conde-Rubio A., Wang H.-C., Martin O.J.F., Boero G., Brugger J. (2022). Precise capillary-assisted nanoparticle assembly in reusable templates. Part. Part. Syst. Charact..

[34] Ni S., Isa L., Wolf H. (2018). Capillary assembly as a tool for the heterogeneous integration of micro- and nanoscale objects. Soft Matter.

[35] Wang X., Hou Y., Yao L., Gao M., Ge M. (2016). Generation, Characterization, and Application of Hierarchically Structured Self-Assembly Induced by the Combined Effect of Self-Emulsification and Phase Separation. J. Am. Chem. Soc..

[36] Chang L., Rissin D.M., Fournier D.R., Piech T., Patel P.P., Wilson D.H., Duffy D.C. (2012). Single molecule enzyme-linked immunosorbent assays. J. Immunol. Methods.

[37] He L., Tessier D.R., Briggs K., Tsangaris M., Charron M., McConnell E.M., Lomovtsev D., Tabard-Cossa V. (2021). Digital immunoassay for biomarker concentration quantification using solid-state nanopores. Nat. Commun..

[38] Huang Q., Li N., Zhang H., Che C., Sun F., Xiong Y., Canady T.D., Cunningham B.T. (2020). Critical Review: Digital resolution biomolecular sensing for diagnostics and life science research. Lab Chip.

